# Isolation of Thermostable Lignocellulosic Bacteria From Chicken Manure Compost and a M42 Family Endocellulase Cloning From *Geobacillus thermodenitrificans* Y7

**DOI:** 10.3389/fmicb.2020.00281

**Published:** 2020-02-26

**Authors:** Lingling Ma, Yuchun Zhao, Limin Meng, Xin Wang, Yanglei Yi, Yuanyuan Shan, Bianfang Liu, Yuan Zhou, Xin Lü

**Affiliations:** ^1^Laboratory of Bioresources, College of Food Science and Engineering, Northwest A&F University, Yangling, China; ^2^State Key Laboratory of Microbial Metabolism, Joint International Research Laboratory of Metabolic & Developmental Sciences, School of Life Sciences and Biotechnology, Shanghai Jiao Tong University, Shanghai, China

**Keywords:** chicken manure compost, *Geobacillus thermodenitrificans*, switchgrass, thermostable, clone and expression, M42 family

## Abstract

The composting ecosystem provides a potential resource for finding new microorganisms with the capability for cellulose degradation. In the present study, Congo red method was used for the isolating of thermostable lignocellulose-degrading bacteria from chicken manure compost. A thermophilic strain named as *Geobacillus thermodenitrificans* Y7 with acid-resident property was successfully isolated and employed to degrade raw switchgrass at 60°C for 5 days, which resulted in the final degradation rates of cellulose, xylan, and acid-insoluble lignin as 18.64, 12.96, and 17.21%, respectively. In addition, GC-MS analysis about aromatic degradation affirm the degradation of lignin by *G. thermodenitrificans* Y7. Moreover, an endocellulase gene belong to M42 family was successfully cloned from *G. thermodenitrificans* Y7 and expressed in *Escherichia coli* BL21. Recombinant enzyme *Cel*-9 was purified by Ni-NTA column based the His-tag, and the molecular weight determined as 40.4 kDa by SDA–PAGE. The characterization of the enzyme *Cel*-9 indicated that the maximum enzyme activity was realized at 50°C and pH 8.6 and, Mn^2+^ could greatly improve the CMCase enzyme activity of *Cel*-9 at 10 mM, which was followed by Fe^2+^ and Co^2+^. Besides, it also found that the β-1,3-1,4, β-1,3, β-1,4, and β-1,6 glucan linkages all could be hydrolyzed by enzyme *Cel*-9. Finally, during the application of enzyme *Cel*-9 to switchgrass, the saccharification rates achieved to 1.81 ± 0.04% and 2.65 ± 0.03% for 50 and 100% crude enzyme, respectively. All these results indicated that both the strain *G. thermodenitrificans* Y7 and the recombinant endocellulase *Cel*-9 have the potential to be applied to the biomass industry.

## Introduction

Energy-related issues and degradation of lignocellulose have attracted widespread attention of researchers, for the sugar and other by-products obtained from lignocellulose degradation possess great economic value and also be important to the development of the green energy industry. However, in terms of lignocellulosic biomass conversion, current technologies are still not suitable for the large-scale applications due to the low efficiency in the destruction of crystallinity and heterogeneity of the raw materials (Sheng et al., 2016). Switchgrass is a warm-season perennial C4 plant and a good source for biofuels conversion with a low planting cost (Vogel et al., 2002; Zhang et al., 2019). Meanwhile, switchgrass is a suitable biofuels feedstock and always acts as a conventional material for the study of lignocellulose degrading (McLaughlin et al., 2006; Wu et al., 2006).

Pretreatment of raw materials is a major bottleneck in the lignocellulose degradation, which always needs a high temperatures conditions (Chen and Qiu, 2010; Mishra and Ghosh, 2017). Physical, chemical, and biological methods are generally used for pretreatment at lab- or industrial-base (Yi et al., 2009), meanwhile, the biological method is also a good approach for the pretreatment, which mainly rely on microorganisms to promote saccharification of the lignocellulose biomass, such as fungi and bacteria. These microbes also secret unique cellulase enzymes that allow the utilization of different forms of cellulose (Saddler, 1982; Gilkes et al., 1991). Moreover, cellulosic microbes and cellulases have a broad research value and the potential to be applied to the industry.

Organic materials can be degraded into low-molecular components in composting process by abundant microorganisms living in compost. Microbes, such as fungi, bacteria, actinomycetes and streptomycetes, were found to have the ability for the degradation of lignocellulose (Varma et al., 2017). Compost microenvironment can serve as a potential resource for the screening of new microbes, metabolites, and gene sequences, as well as the novel cellulose-degrading bacteria. Moreover, studies have been shown that the compost of animal feces contains the bacteria possessing the ability if lignocellulose degradation, such as cow manure (Zang et al., 2017), vermicomposting (Aira et al., 2006), and horse manure (Sheng et al., 2016).

Studies has also been reported that cellulase activities could be detected in animal excrement composts, including cow manure, pig manure, and chicken manure (Dongli et al., 2009). It also found that the chicken manure could be used for the degradation of cellulose, hemicellulose, and lignin by mixing with dairy manure (Rehman et al., 2017). In addition, the normal body temperature of chicken is 41–42°C, which is higher than other ordinary mammals and provides a possibility foundation of the screening of thermostable microbes. Moreover, for the chickens are omnivorous poultry and eat grains, vegetables, and small bugs, the microbes in chicken compost would be more plentiful than that in herbivores. Additionally, chicken manure compost processed with a high fermentation temperature, which provided a good opportunity for the screening of thermostable lignocellulolytic microbes. However, there have no reports about the screening of lignocellulose degradation microbes. Bacteria have more advantages than fungi, e.g. fast growth, excellent pressure resistance and easy genetic manipulation, which makes them highly suitable for the industrial applications. Based on the above reasons, bacteria with the capacity of lignocellulose degradation were screened from chicken manure compost in the present study.

Recent studies on thermophilic microbes have demonstrated the its ability of lignocellulose degradation (Strang et al., 2017; Bibra et al., 2018). Thermophilic microorganisms exhibited many advantages during the industrial applications, for example, the relative higher temperatures can increase hydrolysis rates and improve degradation ratio, as well as save costs. Consequently, this study screened a thermostable lignocellulolytic bacterium from chicken manure compost, which identified and named as *Geobacillus thermodenitrificans* Y7. For the strain Y7 had a strong ability to degrade lignocellulose in switchgrass, it was interesting to explore the lignocellulose degradation enzymes contained in this strain. Based on this consideration, an endocellulase gene was cloned from *G. thermodenitrificans* Y7 in this study. Moreover, an endocellulase *Cel*-9 belonging to M42 family was successfully expressed in *E. coli* BL21. Enzymatic properties of *Cel*-9 were characterized and xylan degradability was also analyzed. Moreover, its application in switchgrass hydrolyzation shows the huge potential application of both the strain and recombinant enzyme.

## Materials and Methods

### Materials

Chicken manure compost was collected from the Jiangsu Tianmiaoli Fertilizer Co., Ltd., Long straw-like switchgrass was air dried before transferred into lab, then washed with water and dried at 60°C in oven, which then crushed using a high-speed pulverizer to 40 mesh and oven-dried for further experiments. The chemicals used in this study were purchased from Kelong Chemical Reagent Co., (China), Aladdin (China), or Sigma (United States). The bacterial genomic DNA extraction and plasmid extraction kits were purchased from OMEGA (United States).

### Thermostable Bacteria Isolation

A total of 1 g of chicken manure compost was added into a selected medium (2.0 g/L CMC-Na, 2.0 g/L (NH_4_)_2_SO_4_, 0.5 g/L MgSO_4_⋅7H_2_O, 1.0 g/L K_2_HPO_4_ at a pH of 7.20) and incubated at 60°C for 48 h with a constant shaking speed of 150 rpm. Then, a serial dilution of the enriched bacterial suspension was spread onto Congo red agar plates containing 20.0 g/L agar within the selected medium. The plates were incubated at 60°C for 72 h, and then different colonies on the plates were selected. “Indices of Relative Enzyme Activity (I_CMC_ = diameter of clearing or halo zone/colony diameter) on Congo red agar plates was used to identify the cellulolytic bacteria and record the endo-cellulase activity (Ventorino et al., 2015; Pennacchio et al., 2018).

### Analyses of 16S rRNA Gene Sequences

The bacteria were cultured in CMC-Na liquid medium for 48 h, and then, the cells were harvested and used for chromosomal DNA extraction by a DNA extraction kit (Sangon Biotech, China). The universal primers 27F and 1492R (Gamcsik et al., 1996) were used to amplify the 16S rRNA gene fragments. The polymerase chain reaction (PCR) reaction mixture was composed of 1 μL DNA template (100 ng), 1 μL upstream primer (10 μM), 1 μL downstream primer (10 μM), 12 μL mixture, and 10 μL double-distilled water to make a final total volume of 25 μL. The PCR conditions were carried out as follows: initial denaturation at 95°C for 5 min; 35 cycles of 94°C for 1 min, 58°C for 1min, and 72°C for 3 min; the final extension at 72°C for 10 min.

Agarose gel electrophoresis was used to confirm the target products, and the PCR products were retrieved for sequencing. The 16S rDNA sequences were confirmed and compared using a BLAST nucleotide searching in NCBI (http://blast.ncbi.nlm.nih.gov/). The sequence data for *G. thermodenitrificans* Y7 were submitted to the GenBank database with accession no. MK355519. The phylogenetic tree was constructed using MEGA 6.0 software.

### Bacterial Growth Curve Measure

An overnight culture of *G. thermodenitrificans* Y7 was diluted to 10^6^ cfu/mL of cells and incubated in CMC-Na single carbon source liquid medium. All cultures were incubated at 60°C for 36 h. Growth was monitored every 2 h by determination the absorbance at 600 nm. Meanwhile, blank medium was used as the zero control.

### Bacteria Hydrolysis of Unpretreated Switchgrass

*Geobacillus thermodenitrificans* Y7 was grown in CMC-Na liquid medium until reaching an OD_600_ of 1.0 at 60°C, 150 rpm. Then, 10% of the strain was inoculated into the switchgrass medium (7.0 g/L switchgrass, 2.0 g/L (NH_4_)_2_SO_4_, 0.5 g/L MgSO_4_⋅7H_2_O, 1.0 g/L K_2_HPO_4_ at natural pH of 5.6, sterilization at 121°C, for 20min). The culture was then incubated at 60°C and 150 rpm; for 5 days. An inoculated sample without adding strains served as the blank. The pH of the cultural liquid was also recorded daily.

### Switchgrass Degradation Analysis

The cultural liquid was filtered through a 0.22 μm water filter, and the amount of total reduced sugar was determined using the DNS method (Tasun et al., 1970). Switchgrass samples were oven-dried at 60°C overnight, and the cellulosic compositional analysis of switchgrass samples was performed by an acid hydrolysis method developed by the National Renewable Energy Laboratory (NREL) (Sluiter et al., 2008; NREL, 2012) to detect degraded low molecular sugars and calculate the amount of cellulose and xylan degradation in liquid and dry mass, respectively. All the results are presented as the average values of three independent experiments with standard distinguish at a significance *p* < 0.05.

### Field-Emission Scanning Electron Microscope (FE–SEM) and GC–MS Analysis of Switchgrass Hydrolysis

Shredded lignocellulosic material degraded by incubation with *G. thermodenitrificans* Y7 for 5 days was inspected by FE-SEM to visually study the decay process (Kuuskeri et al., 2016). GC-MS was used to observe the degradation of switchgrass lignocellulose. Both the FE-SEM and GC-MS analyses followed the methods of Yang (Yang et al., 2018). The controls consisted of cultures with no bacteria. The identification of lignin-degradation–related components was performed by comparing with the NIST library based on retention times (RTs).

### Amplification of the Full-Length Cellulase Gene

A cellulase gene, *Cel*-9, from *G. thermodenitrificans* Y7 was amplified from the genomic DNA by PCR. The gene encoding the cellulase was amplified using primers designed based on the cellulase gene sequence (Supplementary S1) of the strain *G. thermoleovorans* CCB_US3_UF5 (CP003125.1), which was noted as “similar to Cellulase M and related proteins” in NCBI. The gene encoding the cellulase was amplified using PCR with the forward primer 5′-CATG*CCATGG*GCATGG CGAAGTTGGACGAAACGTT (*Nco*I site italic type), reverse primer 5′-CCG*CTCGAG*TTCGTCAAACGTCAGTT GTTTCACT (*Xho*I site italic type) and genomic DNA as a template. Primers were designed by deleting the stop codon that could using 6 × his on the pET-28a(+) vector after *Xho*I site. The final recombinant enzyme contained a His-tag at the C-terminal and two another adding amino acid before his-tag (gene and amino acids sequences were shown in Supplementary S2). Whole sequence of *Cel*-9 ORF region was submit to GenBank Database, and which obtained the accession number of MN907430.

### Cloning and Expression of the Cellulase in *E. coli* BL21

The amplified PCR product was inserted into the corresponding sites of the pET-28a (+) vector (Novagen, Germany) by Takara restriction enzymes (Takara Bio, Shiga, Japan), and the constructed plasmid was transformed into *E. coli* BL21 (DE3) for gene cloning and expression. 1% agarose gel was used to detect the accuracy of the inserted gene, and the PCR products were verified by the sequencing, which ensured its integrity. The resulting cellulase expression plasmid wad sequenced to observe the complete cellulase sequence. In addition, expression of the cellulase was induced with 0.4 mM IPTG when the OD_600_ of the bacterial suspension reached to 0.6 and then further cultured at 25°C overnight.

### Purification of the Recombinant Protein

The cells were collected by centrifuging at 12,000 × *g* at 4°C for 10 min and then suspended in PBS buffer (pH 7.2), which was followed by ultrasonication for 20 min by a SCIENTZ-IID ultrasonic homogenizer (Ningbo Scientz Biotechnology Polytron Technologies Inc., Zhejiang, China). The cell lysates were centrifuged at 8000 × *g* for 30 min at 4°C, and the centrifugation, the supernatant was loaded onto a Ni–NTA His Bind resin column (Novagen, Germany), which was washed with 20 mM imidazole (1 × PBS, 20 mM imidazole, pH 7.2) and then eluted with 250 mM imidazole (1 × PBS, 250 mM imidazole, pH 7.2). Finally, the eluted protein was detected by 12% SDS–PAGE.

### Enzyme Assay of Recombinant Protein

Endocellulase activity (CMCase activity) was measured by the DNS assay with 1% CMC-Na as the substrate based on the method described by Ghose (Ghose, 1987). The reaction mixture containing 50 μL enzyme solution, 50 μL buffer, and 100 μL of 1% CMC-Na prepared in 100 mM phosphate buffer at pH 7.0 was incubated at 50°C for 30 min. The reaction was terminated by adding 300 μL DNS, and then heated at 100°C for 5 min. The absorption of the reaction mixture was measured at 540 nm by a Victor X3 Multimode Plate Reader (PerkinElmer, United States). Reduced sugars of each cultured liquid were assayed by the DNS method (Tasun et al., 1970). One unit (U) of enzyme activity was defined as the amount of enzyme that produced 1 μmol of D-glucose in 1 min under the described assay conditions.

The activity of CMCase was confirmed by a thin layer chromatography (TLC) assay with the substrate CMC-Na. Enzyme action was stopped by heating with boiling water for 5 min, and then, 20 μL of substrate and production sample as well as 2 μL of 1mg/mL glucose were applied to a TLC plate with the running buffer composed of n-butanol: ethanol: water (5:3:1, v/v/v). The color rendering was performed at 85°C for 30 min after spraying with methanol: sulfuric acid (9:1, v/v) (Ishibashi et al., 2012; Bala and Singh, 2017).

### Characterization of the Purified Recombinant Protein

Effect of pH on cellulase activity was determined by enzyme activity determination over a pH range of 2.5–11.0 at 50°C for 30 min in the buffers composed of 50 mM citrate (pH 3.0–6.0), sodium phosphate (pH 6.0–9.0), and glycine-NaOH (pH 9.0–10.0). For the pH stability assay, the enzyme was incubated at 50°C in the different buffers for 30 min without substrate and then measured the enzyme activity.

The effect of the reaction temperature was measured between 0–100°C at 10°C steps at the optimal pH for 30 min. The thermal stability of the enzyme was assessed with pretreatment at different temperatures for 30 min before the enzyme activity was then measured under control conditions.

The effect of metal ions (Na^+^, K^+^, Cu^2+^, Mg^2+^, Zn^2+^, Mn^2+^, Co^2+^, Fe^2+^, Fe^3+^, Ca^2+^, and NH^4+^) at concentrations of 10, 5, and 1 mM, as well as chemicals EDTA, SDS, DTT, and β-Mercaptoethanol (1.0, 0.5, and 0.1%) on the enzyme activity were investigated. After adding various metal ions and chemicals into enzyme action system at room temperature for 30 min, enzyme activities were measured with the standard conditions. The enzyme activity of the control was set as 100%, and the relative activity under each condition was recorded.

The kinetic parameters, *K*m and *V*max, were measured with the substrate (CMC-Na) at 0.1–15.0 mg/mL after incubation with the purified *Cel*-9 cellulase at pH 8.6 and 50°C for 30 min. The data were plotted according to the Lineweaver-Burk method.

The substrate specificity of the purified *Cel*-9 was determined at 1% (w/v) different substrates, with included barley glucan, laminarin, pullulan, maltose, Avicel, xylan from beechwood, filter paper, and CMC-Na. The enzyme activity measured for CMC-Na was set as 100%, and the relative activity for each substrate was recorded.

### Sequence Analysis and Homology Modeling

The obtained DNA sequence was translated into a protein sequence and then analyzed by BLAST based on the National Center for Biotechnology Information (NCBI) database. Multiple sequence alignments were completed by the Clustal Omega website (https://www.ebi.ac.uk/Tools/msa/clustalo/). The molecular weight, theoretical pI, instability index, aliphatic index, and grand average of hydropathicity (GRAVY) were predicted by the website ExPASy (https://web.expasy.org/protparam/). The secondary structure of the recombinant enzyme was predicted according to the online resources at the Bloomsbury Centre for Bioinformatics (http://bioinf.cs.ucl.ac.uk/psipred/). The structure of *Cel*-9 was modeled by the I-TASSER online server (https://zhanglab.ccmb.med.umich.edu/I-TASSER/) (Zhang, 2008; Yang et al., 2015).

### Application in Hydrolyzation of *Cel*-9 in Raw Switchgrass

Raw switchgrass was prepared as showed before. The 50% and 100% crude recombinant enzyme in a pH 8.6 buffer (optimum pH) were used for the saccharification of switchgrass in 5% (w/v) at 50°C for 20 h, and the pretreatment without enzyme was set as blank. Reducing sugar content of the hydrolyzation liquid after centrifugation was measured by the DNS method. The effect of the pretreatment with crude enzyme was recorded and saccharification rate was calculated by the following formula (Dadheech et al., 2018): Saccharification rate (%) = Sugar contents (mg)/Substrate (mg) × 100.

All analyses were conducted in triplicate in this study and the results showed as mean ± sd.

## Results

### Isolation, Identification of Lignocellulolytic Bacteria From Chicken Manure Composting and Growth Characteristics

This study intended to isolate potentially lignocellulose degradation bacteria from chicken compost for industrial applications. Congo red plate method is an important tool for the screening of cellulolytic strain with low-cost and convenient (Sajith et al., 2014). Over 80 bacteria strains were isolated from the chicken manure compost at 60°C and twenty of them displayed a strong ability to hydrolyze CMC-Na. According to the results of I_CMC_ (the ratio of the clearing zone diameter to colony diameter), strain Y7 stood out from these colonies, as the most of the other strains with ratios ranged from 2 to 4 (Supplementary S3). Therefore, the strain Y7 was selected for the further study.

**FIGURE 1:**
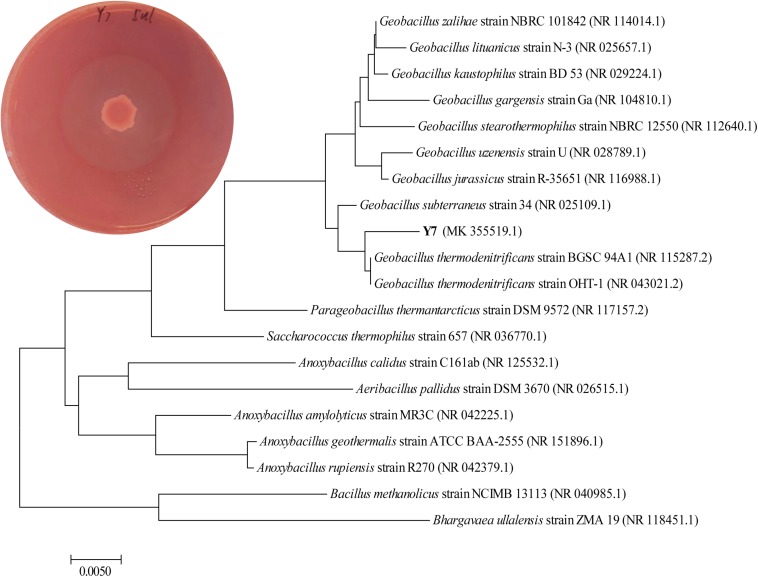
Phylogenetic tree analyzed by 16S rRNA gene sequence with the neighbor-joining methods constructed with the MEGA 6.0. *Geobacillus thermodenitrificans* Y7 achieve GenBank accession number MK355519. Hydrolyzed circle of isolates on the Congo red agar plate of Y7 with single carbon source of CMC-Na was shown on the corner left above.

After sequence alignment and analysis by the BLAST in NCBI database of 16S rRNA gene sequence, the strain Y7 was identified as *G. thermodenitrificans* Y7 with an accession number MK355519 provide by GenBank. It has 99.45% identification percentage with strain *G. thermodenitrificans* OHT-1 and 99.38% identification percentage with strain *G. thermodenitrificans* BGSC 94A1. A neighbor-joining phylogenetic tree of Y7 was shown in Figure 1. In addition, the content of G and C base was high (58.80%) in strain Y7 16S rRNA gene sequence, which implied that Y7 might be a thermophilic strain (Nazina et al., 2001).

**FIGURE 2:**
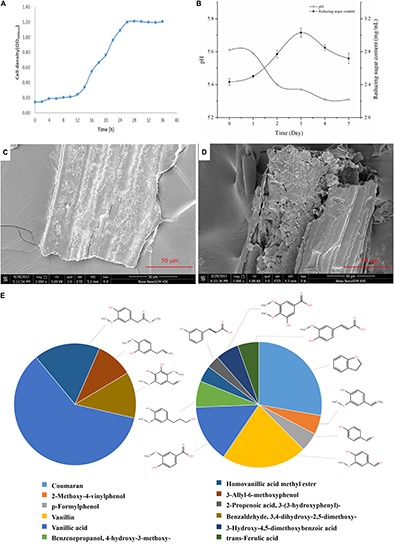
Growth and degradation characters of *Geobacillus thermodenitrificans* Y7. **(A)** Growth profile of Y7 in CMC-Na medium over 36 h of incubation at 60°C; **(B)** PH and total reducing sugar content of culture broth among 5 days by cultured of *G. thermodenitrificans* Y7; **(C,D)** SEM images of switchgrass surface before culture **(C)**, and after 5 days **(D)** cultured by Y7; **(E)** Proportion of absorption peaks area of aromatic compounds in the blank (0 day) (left), and 5th day (right).

The growth curve of *G. thermodenitrificans* Y7 was shown in Figure 2A. The adjustment period was from 0 to 12 h, while the logarithmic phase occurred from 12 to 26 h, and after which it entered a stable phase.

### Degradation of Switchgrass

Unpretreated switchgrass was cultured by Y7, and with the variation of medium pH, reducing sugar content and lignocellulose composition were monitored in the following 5 days. During cultural time, the pH of the cultural broth was remained between 5.3 to 5.6 as shown in Figure 2B, and the total reducing sugar content of the broth reached to the its highest content of 3.03 mg/mL on the 3rd day.

The switchgrass was cultured with strain *G. thermodenitrificans* Y7 for 5 days, and the control consisted of the switchgrass solution without the strain. The liquid and switchgrass composition were shown in Table 1. Over 5 days, the cellulose in the switchgrass underwent 18.64% hydrolysis, while the xylan of hemicellulose was 12.96% degraded, in addition, the acid-insoluble lignin was 17.21% degraded over this period. The acid hydrolyzed sugar content of the culture liquid was also detected to determine the cellulose and xylan degradation relative to the total dry weight, and which stayed more stable between the balance of hydrolyzation and expend caused by the proposed strain.

**TABLE 1:** Chemical compositions of switchgrass degradation by *Geobacillus thermodenitrificans* Y7.

**Cultivation time (days)**	**Weight loss (%)**	**Cultural liquid composition^2^ (% wt/wt)**		**Dry matter composition (% wt/wt)**		
		**Cellulose**	**Xylan**	**Cellulose**	**Xylan**	**Acid-insoluble lignin**
Blank^1^	12.16 ± 0.26	3.39 ± 0.01	1.06 ± 0.09	31.28 ± 0.36	16.44 ± 0.29	19.29 ± 0.10
1	21.59 ± 0.34	3.41 ± 0.03	1.02 ± 0.08	30.10 ± 0.37	16.06 ± 0.38	18.34 ± 0.13
2	22.68 ± 0.67	3.14 ± 0.09	1.00 ± 0.07	28.06 ± 0.26	15.19 ± 0.10	17.27 ± 0.23
3	22.85 ± 0.64	3.39 ± 0.07	1.02 ± 0.08	26.96 ± 0.19	14.63 ± 0.12	16.74 ± 0.13
4	23.22 ± 0.41	3.54 ± 0.07	1.03 ± 0.09	26.14 ± 0.53	14.46 ± 0.16	16.47 ± 0.22
5	24.37 ± 1.35	3.57 ± 0.09	1.11 ± 0.09	25.45 ± 0.34	14.31 ± 0.33	15.97 ± 0.29
Degradation rate				18.64%	12.96%	17.21%

*Values represent mean ± SD (*n* = 3). ^1^Blank was initial switchgrass without adding strain. ^2^Determination of the glucose and xylose content of fermentation broth which had been completely hydrolyzed by acid, and converted into the content of cellulose and xylan.*

Scanning electron microscope imaging was employed, for the effect evaluation on switchgrass by *G. thermodenitrificans* Y7, which characterized the surface of switchgrass before (Figure 2C) and after (Figure 2D) cultivation. By comparing the surface changes of switchgrass before and after hydrolyzed, it indicated that the Y7 strain really hydrolyzed the switchgrass, and which resulted in the increase of roughness and leading to more smaller pieces to the initially smooth surface.

The low molecular weight components produced by the cultural process of switchgrass with *G. thermodenitrificans* Y7 after 5 days were analyzed by GC-MS. The total ion chromatography (TIC) patterns corresponding to the degraded samples and the control were shown in Supplementary S4, and the identification of the aromatic components was listed in Table 2. A total of 13 kinds of aromatic components were both identified in the control and Y7-treated samples. The degradation extent was observed by comparing the peaks that had appeared or disappeared (Kumar et al., 2015) as well as content changes. The increase or decrease in the component contents indicated there was a generation of new degradation products, which implied that the strain Y7 had the ability to degrade the lignin in switchgrass.

**TABLE 2:** Identification of aromatic compounds in control sample and the fifth day degraded switchgrass sample by *G. thermodenitrificans* Y7 as TMS derivatives.

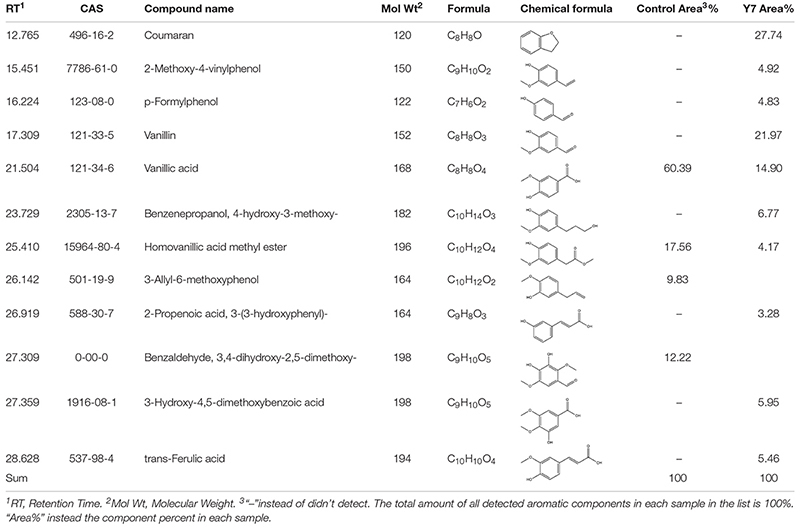

*^1^RT, Retention Time. ^2^Mol Wt, Molecular Weight. ^3^“–”instead of didn’t detect. The total amount of all detected aromatic components in each sample in the list is 100%. “Area%” instead the component percent in each sample.*

Table 2 and Figure 2E showed that the high molecular weight compounds in switchgrass were degraded greatly. Vanillic acid (CAS 121-33-5) and homoyanillic acid methyl ester (CAS 15964-80-4) were partially degraded, while 3-allyl-6-methoxyphenol (CAS 501-19-9), benzaldehyde and 3,4-dihydroxy-2,5-dimethoxy- (CAS 0-00-0) were degraded completely. In addition, eight aromatic components were detected from switchgrass only after treatment with Y7, including coumaran (CAS 496-16-2), 2-methoxy-4-vinylphenol (CAS 7786-61-0), p-formylphenol (CAS 123-08-0), vanillin (CAS 121-33-5), benzenepropanol, 4-hydroxy-3-methoxy- (CAS 2305-13-7), 2-propenoic acid, 3-(3-hydroxyphenyl)- (CAS 588-30-7), 3-hydroxy-4,5-dimethoxybenzoic acid (CAS 1916-08-1), and trans-ferulic acid (CAS 84-74-2). All these aromatic components were of smaller molecular weight and were produced following the degradation of the complex aromatic compounds with high molecular weight. 2-methoxy-4-vinylphenol (CAS7786-61-0) can also be considered an aromatic monomer component, which is the basic component of the lignin structure (Yang et al., 2018). It should be noted that the organic acid contents increased after the degradation processes, including 2-propenoic acid, 3-(3-hydroxyphenyl)- (CAS 588-30-7), 3-hydroxy-4,5-dimethoxybenzoic acid (CAS 1916-08-1), and trans-ferulic acid (CAS 537-98-4), which indicate the primary metabolites might be produced by the microbes during the growth period or intermediate cleavage products from lignin degradation.

### Cloning of the *Cel*-9 Enzyme and Sequence Analysis

**FIGURE 3:**
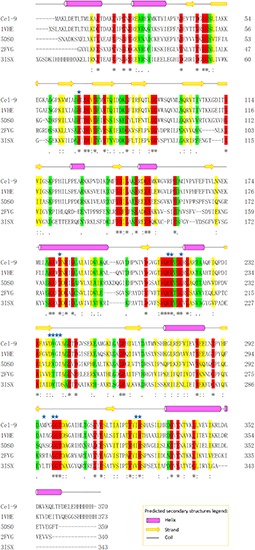
Predicted secondary structure and amino acid sequence alignments for *Cel*-9 and comparison with the characterized M42 endoglucanases. Characterized M42 endoglucanases used for the multiple alignment: *Bacillus subtilis* (PDB: 1VHE) with 75.14% identity, *Thaumarchaeota archaeon* SCGC AB-539-E09 (PDB: 5DS0) with 48.90% identity, *T. maritima* MSB8 (PDB: 2FVG) with 36.34% identity, *T. maritima* (PDB: 3ISX) with 35.57% identity, and *T. maritima* (PDB: 1VHO) with 35.53% identity. Alignments were performed using Clustal Omega. Active site marked by blue stars on top of the aa. Protein secondary structure of *Cel*-9 was predicted by PSIPRED. Helix, strand and coil was showed on top of aa sequence.

Cloning of the *Cel*-9 gene from *G. thermodenitrificans* Y7 into the pET-28a vector was confirmed by sequencing by the universal primers T7/T7er. The translated amino acid sequence was blast in NCBI database. The complete gene sequence of *Cel*-9 was consist of 1113 bp (including 6 × His and another two amino acid), which encoded an enzyme of 370 amino acids (including the His-tag). The predict molecular weight of the recombinant enzyme was 40.42 kDa and the theoretical *pI*-value of *Cel*-9 was 6.06 (https://web.expasy.org/protparam/). The predicted instability index and aliphatic index were computed as 21.42 and 92.76, respectively (Figure 3), which indicated that the *Cel*-9 protein was stable and fat-soluble. The GRAVY for *Cel*-9 was -0.143, which meant that the proposed protein was slightly hydrophilic.

**FIGURE 4:**
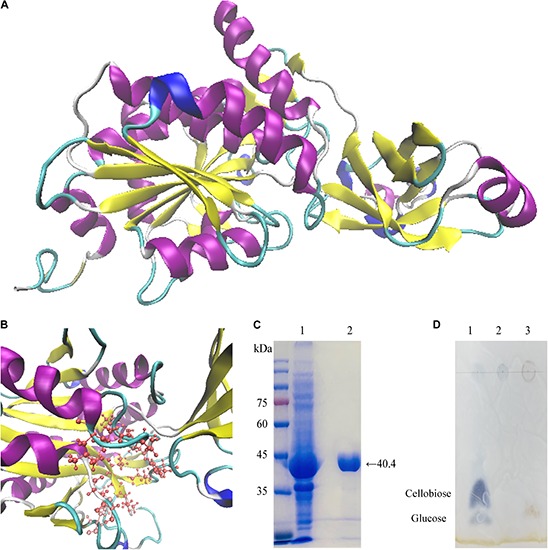
Homology modeling, purification, and end products by TLC plate of *Cel*-9. **(A,B)** predicted structural model of *Cel*-9 with two domains from *Geobacillus thermodenitrificans* Y7 based on structure of aminopeptidase/glucanase from *Bacillus subtilis* (1VHE _A) by I-TASSER **(A)** and activity sites present a pocket structure shown in panel **(B)**. **(C)** SDS–PAGE analysis of purified His-tagged *Cel*-9. Left lane showed protein marker. Lane 1: Crude enzyme extracted from cell of *E. coli* BL21 (DE3) harboring His-tagged *Cel*-9. Lane2: Purified His-tagged *Cel*-9 using a Ni-NTA affinity column by imidazole gradient elution; **(D)** TLC analysis for hydrolysis products of *Cel*-9 use CMC-Na as cellulose substrate. Line 1 was standard contained 2 μL glucose and cellobiose (1 mg/mL), Line 2 was 20 μL 1% CMC-Na substrate, Line 3 was 20 μL reaction products of CMC hydrolysis after 2 h.

Predict 3D structure of *Cel*-9 was showed in Figure 4A of homology modeling by I-TASSER web serve. The Conserved Domain Database at NCBI (https://www.ncbi.nlm.nih.gov/cdd) was used to identify domains in the translated *Cel*-9 sequence. The recombinant protein sequence included two domains: one domain encompassed regions 1–71 and 166–370, and the other domain encompassed region 72–165. It got a good match to the “M42_Frv” domain, which was annotated as “M42 Peptidase, Endoglucanases; Peptidase M42 family, Frv subfamily (cd05656, M42 Peptidase, Endoglucanases)” (Supplementary S5). By searching the PDB (Protein Data Bank) database, the Y7-*Cel*-9 protein sequence was shown to share a 75.14% identity with an aminopeptidase/glucanase homolog from *Bacillus subtilis* (pdb id: 1VHE_A) and a 48.90% similarity with peptidase M42 from *Thaumarchaeota archaeon* (pdb id: 5DS0_A). Y7-*Cel*-9 shared only a 36.34% identity and 35.57% similarity with the endoglucanase from *Thermotoga maritima* (pdb id: 2FVG_A and 3ISX_A). By comparing the similarities of sequence, the presence of conserved amino acid residues which related to the catalytic activity was indicated. The M42 family protein conserved feature residue pattern (H D E E [ED] H) was well conserved in the *Cel*-9 protein sequence, as His68, Asp182, Glu214, Glu215, Asp237, and His325. These five amino acids composed the metal-binding site, which served to bind and align divalent metal ions. It was determined that Mn^2+^ was most likely to bind at this site and improve the enzyme activity. Moreover, compared with similar enzyme sequences and amino acids sequences, the active site residues of *Cel*-9 included His68, Asp182, Glu214, Glu215, Leu218, Asp237, Val238, Gly239, Val240, Met295, Gly298, Gly299, Ile324, and His325 (Figure 4B). In addition, glutamic acid (Glu214) was found in all the aligned sequences as a strictly conserved catalytic residue, which likely acted as a general base for hydrolytic catalysis (Schoehn et al., 2006; Dutoit et al., 2012). Additionally, it was worth noting that there was no similarity to other non-redundant sequences among the carbohydrate-active enzymes referenced in the CAZy database in comparing with other glycoside hydrolase sequences (Park et al., 2010).

### Modeling Studies

For 3D structure prediction, the *Cel*-9 sequence was modeled using I-TASSER (https://zhanglab.ccmb.med.umich.edu/I-TASSER/). Based on similarity searches in the protein database, as the crystal structure of aminopeptidase/glucanase from *Bacillus subtilis* 1VHE Chain A was selected as the template for modeling (Figure 4A). The *Cel*-9 sequence showed a 75.14% identity with the template sequence. Figure 4B presents the active domain within the predicted structure, which formed a pocket-like structure.

### Expression and Purification of the Recombinant Protein

The cloned endoglucanase gene *Cel*-9 was expressed and predicted to encode a recombinant protein of 370 amino acids with a control of an IPTG-inducible T7 promoter. The enzyme *Cel*-9 was expressed after induction with 0.2 mM IPTG when expressed from *E. coli* BL21 (DE3) at 37°C for 5 h. The cells were then harvested by centrifugation and resuspended in a 1 × PBS (pH 7.2) buffer solution. After ultrasonic disruption, crude enzyme solution was collected by centrifugation to removal the precipitates. The crude enzyme was loaded onto a Ni-NTA resin column, and imidazole at 20mM was used to elute hybrid protein, then the 200mM to obtain the purified *Cel*-9 protein. SDS–PAGE analysis indicated a single band for the purified enzyme with a molecular weight of 40.42 kDa (Figure 4C). The activity of the purified recombined protein was 0.024 U/mg.

The TLC analysis of end products by *Cel*-9 used CMC-Na as substrate was shown in Figure 4D. It proved that the *Cel*-9 presented an endocellulase activity that produced oligomers with glucose.

### Influence of pH, Temperature, Metal Ions, and Chemicals on Endoglucanase Activity

**FIGURE 5:**
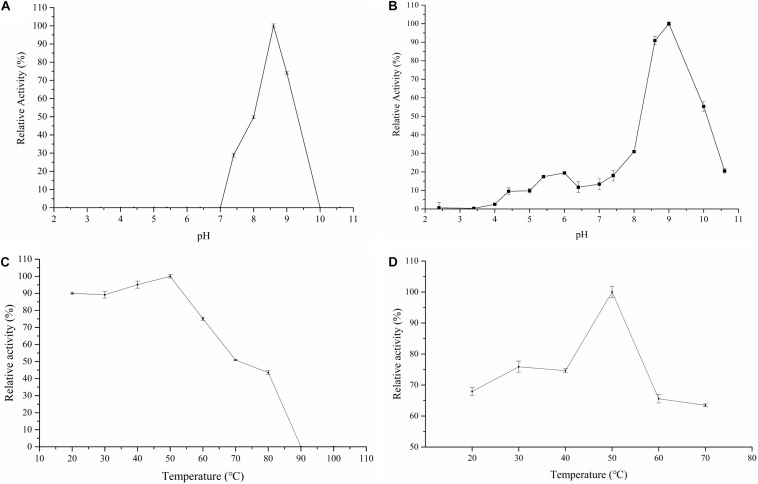
Enzyme character of influence of *Cel*-9 activity. **(A)** influence of pH; **(B)** stability of pH; **(C)** influence of temperature; **(D)** stability of temperature. Three times were repeated for each experiment.

For the purpose to characterize the *Cel*-9 enzyme, the activity of the enzyme at different temperatures (20–100°C) and pH ranges (2.4–10.6) as well as the pH and thermal stability were assessed. The enzyme retained a maximum activity at an alkaline pH of 8.6 (Figure 5A). After incubating at pH 8.6 and 9.0 for 30 min, the enzyme presented a high residual activity, which were 90.87 and 100%, respectively (Figure 5B). As such, a pH range of 8.6–9.0 was determined as the best suitable range for the enzyme to maintain activity. For *Cel*-9, enzymatic reactions under 50°C (Figure 5C) showed the highest activity, reaching 89% or more of this highest activity between 20–50°C, which indicated that the proposed enzyme possessed a wide temperature-active range for hydrolysis, especially at temperatures less than room temperature. Overall, the enzyme remained active over a wide range of temperatures, with the highest activity observed at 50°C after 30 min of incubation (Figure 5D), moreover, which still retained 53.79% activity at 70°C after 30 min.

The effect of metal ions and chemicals on recombinant endocellulase *Cel*-9 is shown in Table 3. It has been reported that different divalent metal ions can influence M42 aminopeptidases (Kumaki et al., 2011; Dutoit et al., 2012). In this study, Mn^2+^ was found to improve CMCase enzyme activity under a 10 mM density, followed by Fe^2+^ and Co^2+^. Additionally, a low density of Cu^2+^ (1 mM) inhibited the enzyme activity. SDS showed an enhancement effect on the enzyme activity, while EDTA had nearly no effect on enzyme activity under 10mM concentration. Other chemicals with different concentrations all showed the inhibition on the enzyme activity with various degree. Kinetic analysis revealed the maximal velocity (*V*max) and Michaelis constant (*Km*) toward CMC-Na were 0.024 μmol/mg/min and 0.310 mg/mL, respectively.

**TABLE 3:** Effect of metal ions and chemicals on the cellulase activity of *Cel*-9.

**Metal ion and chemicals**	**Relative activity (%)^a^**		
	**10mM**	**5mM**	**1mM**
Na^+^(NaCl)	106.72 ± 1.17	103.17 ± 0.85	81.90 ± 1.80
K^+^(KCl)	96.83 ± 3.67	81.53 ± 1.17	84.89 ± 1.17
Cu^2+^(CuSO_4_⋅5H_2_O)	106.72 ± 2.52	89.18 ± 0.32	48.51 ± 1.41
Mg^2+^(MgSO_4_⋅7H_2_O)	144.96 ± 5.04	106.90 ± 2.24	104.66 ± 2.02
Zn^2+^(ZnSO_4_⋅7H_2_O)	66.79 ± 1.17	67.16 ± 0.56	96.27 ± 2.02
Mn^2+^(MnCl_2_⋅4H_2_O)	290.05 ± 4.98	194.94 ± 4.99	96.41 ± 6.89
Co^2+^(CoCl_2_⋅6H_2_O)	147.36 ± 2.21	165.25 ± 6.06	151.50 ± 5.71
Fe^2+^(FeSO_4_⋅7H_2_O)	201.12 ± 1.17	202.80 ± 0.85	141.98 ± 1.41
Fe^3+^(FeCl_3_⋅6H_2_O)	119.96 ± 1.80	195.71 ± 2.76	90.30 ± 0.85
Ca^2+^(CaCl_2_⋅2H_2_O)	133.96 ± 3.37	143.10 ± 1.71	113.62 ± 0.56
NH^4+^(NH_4_Cl)	118.28 ± 2.33	112.69 ± 2.52	87.87 ± 2.02
SDS	130.78 ± 1.41	58.40 ± 1.71	80.97 ± 0.32
EDTA	98.32 ± 0.85	65.67 ± 4.76	71.83 ± 1.17
β-Mercaptoethanol	72.60 ± 1.26(1.0%)	62.47 ± 1.61(0.5%)	58.14 ± 1.22(0.1%)
DTT	83.66 ± 0.79	58.39 ± 2.62	50.08 ± 1.64

*^*a*^Activity without any metal ion or chemicals was set of 100%.*

*Cel*-9 demonstrated a high activity toward barley glucan, laminarin, filter paper, Avicel and xylan from beechwood (Table 4), which indicated that *Cel*-9 possessed the hydrolyzation capacity on the β-1,3-1,4 glucan, β-1,3 glucan, β-1,4, and β-1,6 glucan linkages. Surprisingly, it also demonstrated xylanase activity as well as both β-glucosidase and β-xylosidase activities. In addition, *Cel*-9 had no effect on α-1,4 and α-1,6 glucan linkages, and no activity was also detected against pullulan or maltose.

**TABLE 4:** Substrate specificity analysis of recombinant enzyme and the original enzyme.

**Substrate (1%)**	**Glucan linkage**	**Relative activity (%)^a^**
Barley glucan	β-1,3-1,4 glucan linkage	353.24 ± 6.91
Laminarin	β-1,3 and β-1,6 glucan linkage	429.89 ± 6.56
Pullulan	α-1,4 and α-1,6 glucan linkage	ND
Maltose	α-1,4 glucan linkage	ND
Filter paper		326.47 ± 7.96
Avicel	β-1,4 glucan linkage	245.96 ± 10.78
Xylan from Beechwood	β-1,4 glucan linkage	318.86 ± 4.28
CMC-Na		100.00 ± 3.03

*^*a*^Depending on the substrate, activity was determined under optimal conditions. ND, no detectable activity.*

### Application of *Cel*-9 in Raw Switchgrass

**FIGURE 6:**
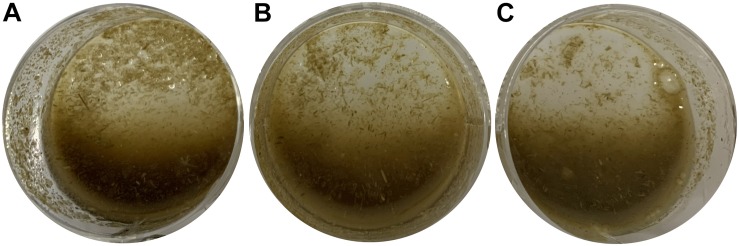
Application of recombinant endocellulase *Cel*-9 into raw switchgrass at 50°C for 20 h. **(A)** as control which only adding equal volume buffer; **(B)** raw switchgrass was adding 50% (v/v) crude enzyme; **(C)** raw switchgrass was adding 100%(v/v) crude enzyme.

Crude enzyme was diluted by pH8.6 buffer to 0, 50, 100% (v/v), which were then mixed with raw switchgrass in 5% (m/v) and incubated at 50°C for 20 h. After hydrolyzation, switchgrass become loosen and light color of degraded products, finally, switchgrass was hydrolyzed into tiny ones and even into flour state. Liquid in system become thick with more low molecular compounds in it (Figure 6). By measuring reducing sugar content in the cultural liquid, the saccharification rate was calculated as 1.81 ± 0.04 and 2.65 ± 0.03% by 50 and 100% crude enzyme, respectively.

## Discussion

Animal manure compost is usually considered as a process for converting waste materials into value-added products and which always composes of four phases: mesophilic, thermophilic, cooling, and maturation (van Bochove et al., 1996). The bio-oxidative transformation process depending on, plentiful microbes, which was the main reason for the high temperatures generated during composting (Sánchez San Fulgencio et al., 2018). As such, it has been confirmed that the compost was a potential source for the isolation of thermophilic and thermotolerant species (Lopez-Gonzalez et al., 2015). Different animal manures contain unique microbes due to the individual foods and digestion processes of the different animals. Cellulose-degrading strains have been identified from different animal feces, which include both fungi and bacteria. Because of the high cellulose content in the manure of cattle, dairy and horse, many studies have been applied for the screening of cellulolytic microorganisms, such as bacteria (Ghosh et al., 2018) and fungi (Zang et al., 2017) from cattle manure, fungi from dairy manure (Wen et al., 2005), fungi (Vandamme et al., 2007), and bacteria (Sheng et al., 2016) from horse manure and bacteria from pig manure (Lee et al., 2010). However, there are few reports on the screening of thermophilic microbes. Besides cellulose-degrading fungi grow temperature is 45°C (Vandamme et al., 2007) and bacteria from horse manure can grow under 60°C (Sheng et al., 2016), other microorganisms almost just grow well under 30–37°C. In addition, there were no reports regarding lignocellulolytic strains isolated from chicken manure, especially the thermophilic bacterium. Chicken manure is usually treated as wastes (Lane, 2008), and has been shown to have the potential to degrade cellulose (Rehman et al., 2017). Additionally, chicken has the higher body-temperature than other animals and generally be feed on various food, include bugs, grain, even fruit and vegetables. All these characters would make their digestive system more plentiful than an herbivore. When the chicken manure was fermented to compost, a thermostable microbe group was formed. This current study was the first time to isolate a thermostable lignocellulolytic bacterium from chicken manure compost with a growth temperature of 60°C, which was named as *G. thermodenitrificans* Y7 and showed potential ability for lignocellulose degradation.

Strain Y7 isolated by the largest clearing zone on Congo red plate. The I_CMC_ of Y7 was as high as 4.6 (Supplementary S3) when the single carbon source of medium was CMC-Na. Compared the diameter of clear zone to other colonies, strain Y7 showed a strong ability to hydrolyze CMC-Na as a representative of cellulose substrate. Further studies indicated that it also possessed well resistant capacity in the low pH environment, which was benefit for the application of Y7 to industry.

Up to now, bacteria possessing high efficiency in lignocellulose degradation remains very few. One reason is that bacteria often grow under 30-37°C and cannot meet the optimum temperature for cellulase working. Another reason is the hydrolyzation rate of bacteria is always lower than fungi and chemical methods generally. In addition, most bacteria were aimed at single composition of lignocellulose, but the degradation of all components of the lignocellulose in a short time is hardly find out. *G. thermodenitrificans* Y7 provide a possibility to enhance the degradation rate. When strain Y7 was utilized into unpretreated switchgrass hydrolyzation, it brought a surprise of its hydrolyzation ability. Switchgrass granule got into finer particles after being hydrolyzed by *G. thermodenitrificans* Y7, moreover, the solution consistency became more diluted when compared to the initial solution. Meanwhile, it also provided a good yield of reducing sugar by cultured with bacteria in few days. The accumulation amount of total reducing sugar content reached to the maximum of 3.03 mg/mL at the 3rd day of cultural, which was equal to 482.86 mg/g of raw switchgrass. It showed higher oligosaccharide yields when compared with the *Ruminiclostridium thermocellum* M3, which was isolated from compost source of horse manure and showed an accumulation of reducing content as 52.7–107.8 mg/g in different biomass (Sheng et al., 2016). The total degradation rate of cellulose, xylan, and acid-insoluble lignin was 18.64, 12.96, and 17.21%, respectively, which indicated that the Y7 strain had a strong hydrolyzing ability to the switchgrass. Although Strain Y7 perform not same well with some fungi (for example, *Penicillium expansum*) (Tao et al., 2009), it displayed excellent performance when compared with other bacteria. For example, the *Rhizobium sp.* strain YS-1r hydrolyzed switchgrass (over 4 months at 30°C) and led to the degradation of glucan (19.33%), xylan (6.56%), and acid-insoluble lignin (15.06%) (Jackson et al., 2017). Besides, when fungi mixture (*Aspergillus niger* and *Trichoderma viride*) cultured unpretreated corn straw, cellulose degradation rate reached around 40% need almost more than 35 days (Youzhou et al., 2015). On the contrary, when strain Y7 was cultured with switchgrass, the cellulose degradation rate reached 18% only need 7 days. Therefore, thermophilic strain *G. thermodenitrificans* Y7 in this study was a potential bacterium for the lignocellulose degradation.

Scanning electron microscope observations indicated that crude enzymes secreted by *G. thermodenitrificans* Y7 had the ability to degrade the biomass efficiently. After treatment with Y7, the length and width of the switchgrass were substantially decreased. Similar structural changes were also observed in the hydrolyzed wheat straw (Kshirsagar et al., 2016) and switchgrass (Wang et al., 2017). The SEM investigations observed a good growth and attachment of *G. thermodenitrificans* Y7 on the surface of the switchgrass. For cellulose degradation, lignin is always considered as a key factor that protects cellulose from hydrolysis, as lignin is wrapped on the outside and reduces the contact between cellulose and the microbes/enzymes (Sun et al., 2014). As is known, lignin is a complex heteropolymer that is difficult to degrade and convert (Brown and Chang, 2014). Removal or degradation of the lignin would make cellulose easier to hydrolyze by bacteria or enzymes due to the increasing of the available surface area. In this study, lignin degradation was observed and determined by the production of monomer benzene compounds and other low-molecular-weight compounds that was related to the lignin degradation (Chen et al., 2012). Results of the GC-MS analysis indicated that the lignin in switchgrass had really been degraded by *G. thermodenitrificans* Y7.

During the pretreatment of switchgrass by the strain *G. thermodenitrificans* Y7, the pH of the culture liquid decreased from 5.61 to 5.31 and then stabilized, which was probably due to the accumulation of organic acids and other acidic metabolic products. Additionally, the results of the GC-MS analysis showed that the organic acid content increased after lignin degradation, which could also be an important reason for the decrease of pH. It also implied that bio-acids provide an acidic environment which would help the degrading switchgrass by enzymes. Meanwhile, the observed increase in the reducing sugar content might be attributed to the low pH that would prevent microbes from further utilizing of glucose or other carbon sources, and similar observations having been reported about *R. thermocellum* (Sheng et al., 2016). All these phenomena indicate that *G. thermodenitrificans* Y7 could be resistant to a low acid environment, which would expand the scope of its industrial application. Besides pH, high hydrolysis temperatures were another key factor in the degradation process for which promoted the activities of lignocellulose-degrading enzymes. Moreover, as the high aerobic was necessary for the cultivation period, the shaking during hydrolyze was essential, and it also improved the contact area between the microbes/enzymes and substrate. The crude enzyme produced by *G. thermodenitrificans* Y7 showed a potential hydrolytic ability.

For the efficiency degradation of strain Y7, it was worth to explore the lignocellulose-degrading enzymes secreted from it. By searching related gene from whole genome of *G. thermodenitrificans* and similar species strains in NCBI database. Fortunately, a target sequence which note “similar to Cellulase M and related proteins” was successfully cloned and expressed. It has been showed that *Cel*-9 amino acid sequence belonged to the M42 family (Supplementary S5) according to the domain analysis. Identification percentage was 97.79% when compared the amino acid sequence in reference sequence database. By the blast of *Cel*-9 amino acid sequence in NCBI, it shared 75.14% with aminopeptidase (pdb id: 1VHE_A) from *Bacillus subtilis*, which indicated that it contained a “M42 peptidase, endoglucanase” region. And the second highest identification percentage was 48.90%, which was another M42 family peptidases from *Thaumarchaeota archaeon* (pdb id: 5DS0_A). CMCase activity was detected for the recombinant *Cel*-9 and which was subsequently characterized. The recombinant *Cel*-9 showed a maximal activity at 50°C, and nearly 90% activity was remained at lower temperatures (20-50°C), which suggested that it possessed a wide active temperature range. *Cel*-9 was shown to be an ion-metal enzyme, and the Mn^2+^ improved the enzyme activity at a density of 10 mM, while Zn^2+^ inhibited the enzyme activity at different densities. Chemicals did not inhibit the enzyme activity at different concentration, but it was interesting that SDS increased enzyme activity under 10mM. Meanwhile, the thermostable cellulase isolated from the *Cel*-9 sample displayed maximal activity at a pH of 8.6, which would be beneficial in biotechnology and industrial applications. Some other M42 family enzymes have also been shown to possess cellulase activity (Maiti et al., 2017), which suggested the possibility that M42 family enzymes might possess different hydrolysis effects on different substrates with suitable metal ions assistant. Assessment of the substrate specificity also showed that *Cel*-9 had activity toward both β-glucosidase and β-xylosidase, which might be the main reason for the high efficiency in lignocellulose hydrolyzation of the strain for the degradation of cellulose and hemi-cellulose (mainly xylan) same time. Moreover, crude enzyme utilized into raw switchgrass also suggested its degradation ability in lignocellulose.

## Conclusion

The acid-resistant thermophilic cellulolytic bacteria *G. thermodenitrificans* Y7 was isolated from chicken manure compost in the present study, which had the ability to efficiently degrade unpretreated switchgrass. As a M42 family cellulase, *Cel*-9 was successfully cloned and expressed, and which presented an alkaline CMCase activity within a broad temperature region. In addition, the catalytic metal ion Mn^2+^ at 10 mM increased the catalytic efficiency of the proposed enzyme. Meanwhile, *Cel*-9 performed well in the degradation of raw switchgrass. Consequently, all the results suggested that both *G. thermodenitrificans* Y7 and *Cel*-9 had a great economic potential for the application to the biomass industry.

## Data Availability Statement

The datasets generated for this study can be found in the GenBank databases Accession No. MK355519.

## Author Contributions

LMa and XL designed the experiments, conducted the study, performed the statistical analysis, and drafted the manuscript. YCZ helped to do the experiments of isolation and lignocellulose hydrolyzation. LMe helped to do the experiments of the character of enzyme de analyze. XW and YY helped to analyze these data and draft the manuscript. YS, BL, and YZ participated in the design of the study and revisions of the manuscript. All authors read and approved the final manuscript.

## Conflict of Interest

The authors declare that the research was conducted in the absence of any commercial or financial relationships that could be construed as a potential conflict of interest.
