# Supplementary material for: Isolation of Thermostable Lignocellulosic Bacteria From Chicken Manure Compost and a M42 Family Endocellulase Cloning From Geobacillus thermodenitrificans Y7
Source: Front Microbiol. 2020 Feb 26;11:281. doi: 10.3389/fmicb.2020.00281 (PMC7054444; doi:10.3389/fmicb.2020.00281)
Supplement: Supplementary file 1 [file Data_Sheet_1.docx]

***Applied Microbiology and Biotechnology***

**Isolation of thermostable lignocellulosic bacteria from chicken manure compost and a M42 family endocellulase cloning from Geobacillus thermodenitrificans Y7**

Lingling Ma^1^, Yuchun Zhao^1,2^, Limin Meng^1^, Xin Wang^1^, Yanglei Yi^1^, Yuanyuan Shan^1^, Bianfang Liu^1^, Yuan Zhou^1^, Xin Lü^1^*

^1^ *Lab of Bioresources, College of Food Science and Engineering, Northwest A&F University, Yangling, Shaanxi Province 712100, China*

^2^ *State Key Laboratory of Microbial Metabolism, Joint International Research Laboratory of Metabolic & Developmental Sciences, and School of Life Sciences and Biotechnology, Shanghai Jiao Tong University, Shanghai 200030, China*

***Correspondence:**

Tel./Fax: +86-029-87082985.

E-mail: [xinlu@nwsuaf.edu.cn](mailto:xinlu@nwsuaf.edu.cn)

**Supplementary 1**

Template gene sequence of primers designing of Cel-9 which searched from NCBI database.

DEFINITION Geobacillus thermoleovorans CCB_US3_UF5, complete genome.

ACCESSION CP003125 REGION: 2807136..2808224

CDS complement (1..1089)

/locus_tag="GTCCBUS3UF5_30560"

/note="similar to Cellulase M and related proteins

COG1363;

similar to Putative aminopeptidase ysdC of root UniRef

RepID=YSDC_BACSU"

/codon_start=1

/transl_table=11

/product="aminopeptidase ysdC"

/protein_id="AEV20358.1"

*Sequence*

ATGGCGAAGTTGGACGAAACGTTGACGATGCTGAAAGCGCTGACCGATGCGAAAGGCGTCCCGGGCAATGAGCGGGAAGCGCGCGATGTGATGAAGACATACATAGCTCCGTATGCGGATGAAGTGACAACGGATGGCCTCGGCAGCTTGATCGCCAAAAAAGAAGGGAAATCGGGCGGGCCGAAAGTGATGATCGCCGGCCATTTGGATGAAGTCGGCTTTATGGTGACGCAAATCGACGACAAAGGATTCATCCGCTTCCAAACGCTCGGCGGCTGGTGGAGCCAAGTGATGCTCGCCCAGCGCGTGACGATCGTGACGAAAAAAGGCGACATCACCGGCGTCATCGGTTCGAAGCCGCCGCACATTCTGCCGCCGGAGGCGCGCAAAAAACCGGTGGAAATCAAAGATATGTTCATCGACATCGGCGCGACAAGCCGCGAGGAAGCGATGGAGTGGGGCGTCCGCCCGGGCGATATGATCGTGCCGTATTTTGAATTTACGGTATTGAACAATGAAAAAATGCTGCTCGCGAAAGCATGGGACAACCGGATCGGCTGTGCGGTCGCCATCGATGTGCTCAAGCAGCTGAAAGGCGTCGACCATCCAAACACGGTATACGGCGTCGGCACGGTGCAGGAAGAAGTCGGCTTGCGCGGCGCGCGCACGGCCGCCCAATTCATTCAGCCAGATATCGCGTTTGCTGTTGACGTCGGCATTGCCGGCGACACGCCGGGGGTGTCGGAAAAAGAAGCGATGGGCAAACTCGGCGCCGGCCCGCACATCGTCTTGTACGACGCAACGATGGTGTCGCACCGCGGCTTGCGCGAATTTGTCATCGAAGTGGCGGAAGAGCTCAACATTCCGTACCATTTTGATGCCATGCCAGGCGGCGGTACGGACGCGGGAGCGATTCACTTAACCGGCATCGGCGTTCCGTCGCTCACGATTGCCATCCCGACGCGCTACATCCACTCGCACGCCGCCATTTTGCACCGCGACGACTACGAAAACACGGTCAAGCTGCTTGTTGAGGTGATCAAACGGCTTGACGCCGACAAAGTGAAACAACTGACGTTTGACGAATAA

**Supplementary 2**

Recombinant *Cel*-9 whole gene sequence and amino acids sequence. 6×His and another two more amino acids are marked out underline.

>Cel-9 ORF gene sequence

ATGGCGAAGTTGGACGAAACGTTAACAATGCTGAAAGCGTTGACGGATGCAAAAGGTGTCCCGGGCAATGAACGGGAAGCGCGCGAAGTGATGAAAACATACATAGCCCCATATGCGGACGAAGTAACGACCGACGGTCTCGGCAGCTTGATCGCCAAAAAAGAAGGAAAAGCTGGCGGACCGAAAGTCATGATTGCCGGCCATTTGGACGAAGTCGGCTTTATGGTCACGCAAATCGATGATAAAGGATTCATCCGCTTCCAAACGCTCGGCGGCTGGTGGAGCCAAGTGATGCTCGCCCAACGAGTGACCATCGTAACGAAAAAGGGCGACATCACCGGTGTGATCGGTTCGAAGCCGCCGCACATTTTGCCGCCGGAGGCGCGCAAAAAACCGGTTGATATTAAAGATATGTTCATTGACATCGGTGCGAAGAGCCGTGACGAAGCGATGAAGTGGGGCGTCCGCCCAGGCGATATGATCGTACCGTATTTTGAGTTTACCGTGTTGAACAATGAAAAAATGCTGTTAGCCAAAGCGTGGGACAACCGGATCGGCTGTGCGATCGCCATTGATGTGCTCAAGCAGCTGAAAGGCGTTGACCACCCGAACACGGTGTATGGTGTCGGCACGGTACAGGAAGAAGTCGGTTTGCGCGGGGCGCGCACGGCTGCCCAATTCATTCAACCGGACATCGCTTTTGCCGTTGACGTCGGCGTGGCAGGCGATACGCCAGGTGTCTCGGAAAAAGAAGCGATGGGCAAGCTCGGCGCCGGCCCGCACATTGTCCTATACGATGCAACGATGGTGTCGCATCGCGGTTTGCGCGAATTTGTCATCGAAGTGGCGGAAGAGCTGAACATTCCGTATCACTTTGACGCCATGCCAGGCGGCGGCACGGACGCGGGGGCGATTCATTTAACCGGCAGCGGTGTCCCGTCACTGACGATCGCCATTCCAACCCGCTACATCCATTCGCATGCTTCCATTTTGCATCGCGATGACTATGAAAACACGGTCAAGCTATTAGTCGAAGTCATTAAACGGCTTGATGCTGATAAAGTGAAACAACTGACGTTTGACGAACTCGAGCACCACCACCACCACCACTGA

>Cel-9 amino acids sequence

MAKLDETLTMLKALTDAKGVPGNEREAREVMKTYIAPYADEVTTDGLGSLIAKKEGKAGGPKVMIAGHLDEVGFMVTQIDDKGFIRFQTLGGWWSQVMLAQRVTIVTKKGDITGVIGSKPPHILPPEARKKPVDIKDMFIDIGAKSRDEAMKWGVRPGDMIVPYFEFTVLNNEKMLLAKAWDNRIGCAIAIDVLKQLKGVDHPNTVYGVGTVQEEVGLRGARTAAQFIQPDIAFAVDVGVAGDTPGVSEKEAMGKLGAGPHIVLYDATMVSHRGLREFVIEVAEELNIPYHFDAMPGGGTDAGAIHLTGSGVPSLTIAIPTRYIHSHASILHRDDYENTVKLLVEVIKRLDADKVKQLTFDELEHHHHHH

**Supplementary 3**

**Table S3**

**Ratio of diameter of transparent circle to colony diameter**

| Strain no. | Clearing zone diameter/mm | Colony diameter/mm | I_CMC_ |
| --- | --- | --- | --- |
| Y7 | 18.5 | 4 | 4.6 |
| Y19 | 15 | 4 | 3.8 |
| Y16 | 12 | 3.5 | 3.4 |
| Y13 | 11.5 | 3.5 | 3.3 |
| Y1 | 13 | 4 | 3.3 |
| Y12 | 13 | 4 | 3.3 |
| Y18 | 13 | 4 | 3.3 |
| Y4 | 12 | 4 | 3.0 |
| Y9 | 12 | 4 | 3.0 |
| Y15 | 14 | 5 | 2.8 |
| Y2 | 12 | 4.5 | 2.7 |
| Y3 | 8 | 3 | 2.7 |
| Y6 | 13 | 5 | 2.6 |
| Y11 | 13 | 5 | 2.6 |
| Y5 | 10 | 4 | 2.5 |
| Y8 | 15 | 6 | 2.5 |
| Y14 | 12 | 5 | 2.4 |
| Y10 | 14 | 6 | 2.3 |
| Y17 | 13 | 6 | 2.2 |
| Y20 | 13 | 7 | 1.9 |

**Supplementary 4**

(a) TIC of control


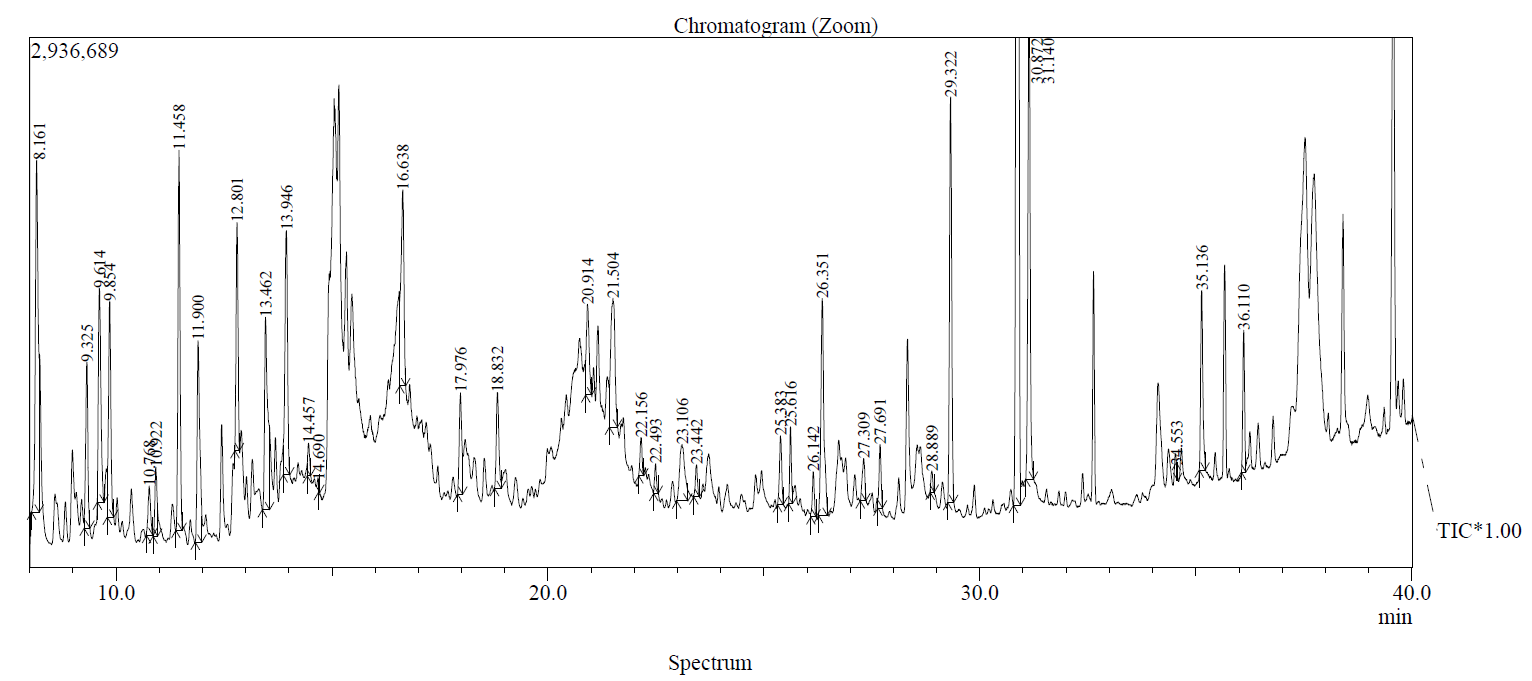


(b) TIC of Y7


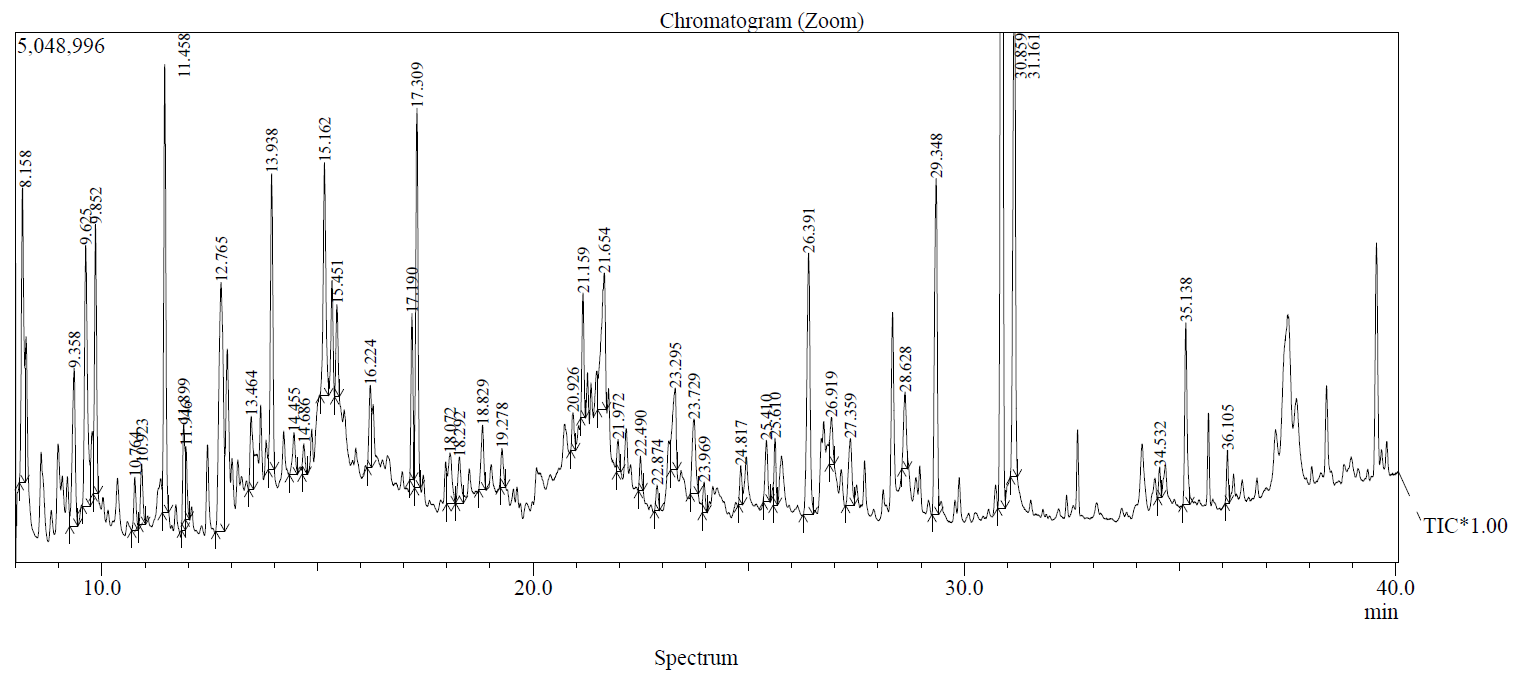


**Figure S4**

The total ion chromatogram (TIC) corresponding to aromatic compounds from control and Y7 treated samples.

**Supplementary** 5


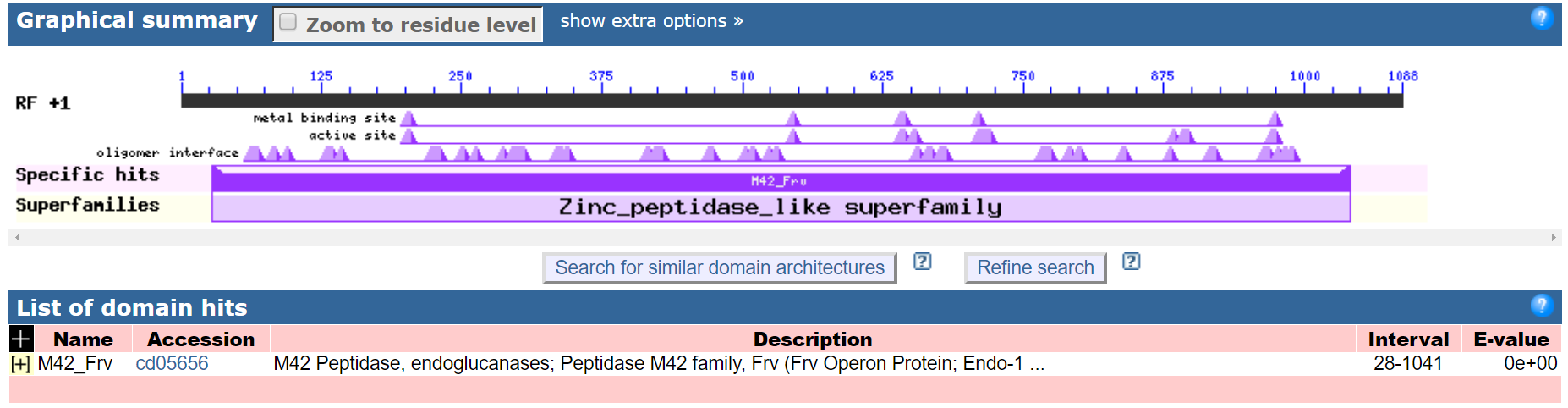


**Figure S5**

The protein sequence of Y7- cellulase hit the M42_Frv domain, which is annotated as the “M42 Peptidase, Endoglucanases; Peptidase M42 family, Frv subfamily (cd05656, M42 Peptidase, Endoglucanases)”.
